# Transjugular Intrahepatic Portosystemic Shunt: Devices Evolution, Technical Tips and Future Perspectives

**DOI:** 10.3390/jcm12216758

**Published:** 2023-10-25

**Authors:** Dario Saltini, Federica Indulti, Tomas Guasconi, Marcello Bianchini, Biagio Cuffari, Cristian Caporali, Federico Casari, Francesco Prampolini, Marco Senzolo, Antonio Colecchia, Filippo Schepis

**Affiliations:** 1 Division of Gastroenterology, Azienda Ospedaliero-Universitaria di Modena, and University of Modena and Reggio Emilia, 41121 Modena, Italy185803@studenti.unimore.it (F.I.); 196482@studenti.unimore.it (T.G.); bianchini.marcello@aou.mo.it (M.B.); cuffari.biagio@aou.mo.it (B.C.); antonio.colecchia@unimore.it (A.C.); 2 Division of Radiology, Azienda Ospedaliero-Universitaria di Modena, and University of Modena and Reggio Emilia, 41121 Modena, Italy; caporali.cristian@aou.mo.it (C.C.);; 3 Multivisceral Transplant Unit-Gastroenterology, Department of Surgery, Oncology and Gastroenterology, Padua University Hospital, 35128 Padua, Italy; marco.senzolo@aopd.veneto.it

**Keywords:** portal hypertension, transjugular intrahepatic portosystemic shunt (TIPS), stent technology, patency, complications, personalized medicine

## Abstract

Portal hypertension (PH) constitutes a pivotal factor in the progression of cirrhosis, giving rise to severe complications and a diminished survival rate. The transjugular intrahepatic portosystemic shunt (TIPS) procedure has undergone significant evolution, with advancements in stent technology assuming a central role in managing PH-related complications. This review aims to outline the progression of TIPS and emphasizes the significant influence of stent advancement on its effectiveness. Initially, the use of bare metal stents (BMSs) was limited due to frequent dysfunction. However, the advent of expanding polytetrafluoroethylene-covered stent grafts (ePTFE-SGs) heralded a transformative era, greatly enhancing patency rates. Further innovation culminated in the creation of ePTFE-SGs with controlled expansion, enabling precise adjustment of TIPS diameters. Comparative analyses demonstrated the superiority of ePTFE-SGs over BMSs, resulting in improved patency, fewer complications, and higher survival rates. Additional technical findings highlight the importance of central stent placement and adequate stent length, as well as the use of smaller calibers to reduce the risk of shunt-related complications. However, improving TIPS through technical means alone is inadequate for optimizing patient outcomes. An extensive understanding of hemodynamic, cardiac, and systemic factors is required to predict outcomes and tailor a personalized approach. Looking forward, the ongoing progress in SG technology, paired with the control of clinical factors that can impact outcomes, holds the promise of reshaping the management of PH-related complications in cirrhosis.

## 1. Introduction

Portal hypertension (PH) is defined from a hemodynamic standpoint as a pathological increase in the portosystemic pressure gradient (PSPG), which represents the pressure difference between the portal vein (PV) and the inferior vena cava (IVC). In the context of cirrhosis, PH has a significant impact on disease progression and prognosis. Normal gradient values range from 1 to 5 mmHg, while values between 6 and 9 mmHg indicate pre-clinical PH. 

The development of clinically significant portal hypertension (CSPH), defined as a PSPG equal to or greater than the threshold of 10 mmHg, leads to the formation of portosystemic collaterals, including esophageal/gastric varices [1]. When this stage is reached, decompensation events such as variceal bleeding, tense ascites, and hepatic encephalopathy (HE) commonly occur. Decompensated cirrhosis is linked to a severely worsened prognosis, reducing median survival from over 12 years in the compensated state to approximately 2 years [2]. 

Controlling PH has been shown to independently reduce the incidence of PH-related complications and death [3,4], providing the rationale for therapies aimed at lowering portal pressure.

Transjugular intrahepatic portosystemic shunt (TIPS) is a minimally invasive interventional radiology procedure known for its remarkable efficacy in reducing PSPG. During a TIPS procedure, a stent is placed to create a low-resistance intrahepatic channel connecting a branch of the PV to a hepatic vein (HV), serving as a side-to-side portocaval shunt, which redirects the portosystemic collaterals blood flow into the systemic circulation [5].

As placement techniques have advanced and stent technology has improved, TIPS has demonstrated its superiority over the standard of care in preventing variceal rebleeding [6,7,8] and the recurrence of difficult-to-treat ascites/hepatic hydrothorax [9,10,11,12]. Current evidence also suggests that TIPS holds promise for novel indications, including non-malignant PV thrombosis [13], Budd-Chiari syndrome [14], PH complications associated with portosinusoidal vascular disorders (PSVD) [15], and “pre-operative TIPS” for high-risk surgery candidates with CSPH [16]. 

Despite advancements in intraoperative techniques and clinical management, shunt creation remains associated with complications that can potentially limit or nullify its benefits. Some innovations, such as the “underdilation positioning strategy” and the “controlled expansion” technology in the latest generation of endoprostheses, have shown encouraging results in enhancing the performance of TIPS.

**Figure 1:**
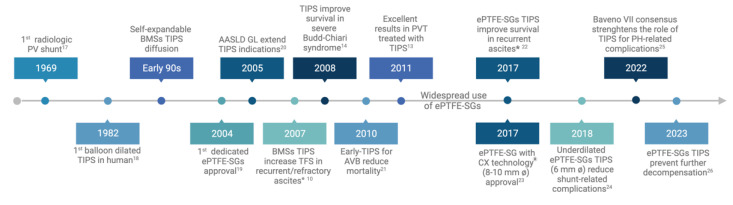
Milestones in TIPS development [10,13,14,17,18,19,20,21,22,23,24,25,26]. (°) = International Ascites Club definition of recurrent and refractory ascites; (*) = recurrent ascites defined by the performance of at least two large-volume paracenteses within a minimum interval of 3 weeks. Abbreviations: AASLD, American Association for the Study of Liver Diseases; AVB, acute variceal bleeding; BMSs, bare metal stents; CX, controlled expansion; ePTFE-SGs, expanded polytetrafluoroethylene-stent grafts; GL, guidelines; PH, portal hypertension; PV, portal vein; TFS, transplant-free survival; TIPS, transjugular intrahepatic portosystemic shunt. (Created with BioRender.com).

This review aims to trace the milestones in the development of TIPS, with a particular emphasis on the role that the technical evolution of stents has played in establishing TIPS as a central therapy for managing complications of PH (Figure 1). By synthesizing the available evidence from relevant studies, we aim to identify both the strengths and weaknesses of this procedure and chart a path for its further evolution.

## 2. Device Evolution for TIPS Creation

TIPS, as originally introduced in an animal model by Rösch et al. in 1969, involved the placement of a silicone-coated spring coil to ensure patency for up to two weeks [17]. Subsequent experiments in animal models continued, and in the late 1970s, Burgener and Gutierrez established shunt tracts in dogs with PH by using balloon dilatation of the parenchymal tract [27]. Although they successfully normalized the portal pressure, occlusion typically occurred within one week. 

**Figure 2:**
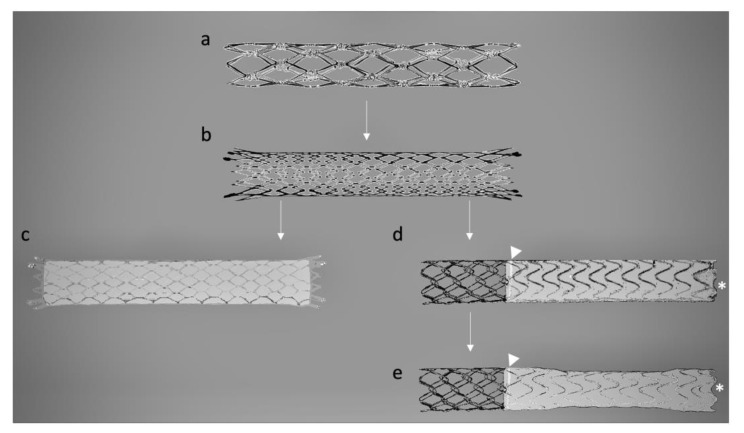
Schematic illustration of the evolution of TIPS devices from the BMSs era to the ePTFE-SGs with controlled expansion technology. (**a**) Palmaz^®^ (Cordis, Miami, FL, USA), a balloon-expandable stainless steel stent; (**b**) Wallstent^®^ (Boston Scientific, Marlborough, MA, USA), a self-expandable BMS made of nickel–cobalt–titanium–steel alloy, with a braided closed-cell design; (**c**) Fluency^®^ (Angiomed GmbH, a subsidiary of C.R. Bard, Inc., Karlsruhe, Germany), a fully covered grid-like stent composed of a biocompatible nickel–titanium alloy, wrapped internally and externally in ePTFE, with 2 mm of bare regions and two radiopaque titanium markers for imaging purposes at both extremities; (**d**) VIATORR^®^ (W.L. Gore & Associates in Phoenix, AZ, USA), a self-expandable dedicated nitinol stent made of a 4 to 8 cm portion covered with ePTFE on the inside and a bare 2 cm long PV portion. A circumferential radiopaque gold band (arrowhead) marks the transition between the covered and uncovered portions and an additional radiopaque gold marker (*) is embedded at the trailing edge of the device; (**e**) VIATORR^®^ Endoprosthesis with Controlled Expansion (W.L. Gore & Associates, Phoenix, AZ, USA), analogous to the VTS with an additional outer constraining balloon-expandable sleeve on the lined region of the stent. Abbreviations: BMSs, bare metal stents; ePTFE, expanded polytetrafluoroethylene; PV, portal vein; TIPS, transjugular intrahepatic portosystemic shunt.

In 1982, Colapinto and Gordon made the first attempt to apply this technique to human patients, involving 20 individuals [18]. Unfortunately, the outcomes were suboptimal, with most patients experiencing rebleeding and there were nine deaths within the first month. A few years later, Palmaz introduced the first stainless steel wire woven mesh stent placed around an angioplasty balloon (Figure 2a) in cirrhotic dog livers, an innovation that significantly improved the patency [28].

Stents used in TIPS procedures need specific mechanical properties, including high elasticity for expansion, strength to withstand liver stiffness, wear resistance, and good biocompatibility to reduce the risk of thrombosis and intimal hyperplasia, which can lead to TIPS dysfunction [29]. The initial generation of vascular stents primarily comprised bare metal stents (BMSs) made of biomedical metals or alloys. For instance, the Palmaz^®^ stainless steel stent (Cordis, Miami, FL, USA), renowned for its robust mechanical strength and corrosion resistance, had reduced flexibility and a potential for complications. Similarly, the nickel–cobalt—titanium–steel alloy employed in the Wallstent^®^ (Boston Scientific, Marlborough, MA, USA) (Figure 2b) and the nickel–titanium (nitinol) alloy used in the Luminexx^®^ (Bard Inc., New Providence, NJ, USA), Zilver^®^ (Cook Medical, Bloomington, IN, USA), and Smart Control^®^ (Cordis, Miami, FL, USA) stents displayed commendable biocompatibility and corrosion resistance. Notably, they possessed unique shape memory properties and elasticity, enabling self-expansion. Nitinol stents, in particular, could undergo significant deformation and return to their original shape or nominal diameter. This innovation marked a significant milestone and facilitated the widespread adoption of the TIPS procedure [30].

In the era of BMSs, shunt dysfunction emerged as a frequent and severe complication of TIPS procedures [31,32,33]. It was often linked to acute thrombosis, pseudointimal hyperplasia resulting from bile leakage, or intimal hyperplasia in the outflow HV [34,35]. This dysfunction resulted in the gradual development of stenosis or occlusion, thereby restricting the long-term effectiveness of TIPS and largely confining its application as a rescue therapy or as a bridge to liver transplantation [36]. 

Prophylactic anticoagulation effectively prevents acute thrombosis of BMS according to the RCT by Sauer et al. [37], which found that the use of phenprocoumon, an anticoagulant, was linked to a reduced incidence of complete occlusion within the first three months following TIPS placement. However, that study showed that it did not significantly affect the incidence of long-term stenosis, which has a different pathogenesis.

To prevent intimal proliferation, various materials, including silicone [38], urethane polycarbonate [39], and polyethylene terephthalate [40], were used to coat stents used for TIPS. However, these coatings did not consistently demonstrate superior patency compared to BMSs [41]. 

In the late 1990s, stents coated with expandable polytetrafluoroethylene (ePTFE), which has minimal permeability to bile and mucin, showed remarkable and prolonged patency due to the absence of neointimal proliferation [42,43,44,45]. This event marked another important milestone in the use of TIPS.

In 2004, the VIATORR^®^ (VTS), developed by W.L. Gore & Associates in Phoenix, AZ, USA, became the first dedicated self-expandable nitinol ePTFE-covered stent graft (ePTFE-SG) to receive FDA approval [19], representing a significant advancement in terms of improved patency in Western countries [46]. Alternatively, in countries where the VTS was not available, non-dedicated ePTFE-SGs, such as Fluency^®^ (Angiomed GmbH, a subsidiary of C.R. Bard, Inc.), primarily designed for treating peripheral vascular diseases, were adapted for TIPS procedures. These ePTFE-SGs have different designs; the VTS features a self-expanding nitinol stent skeleton with a bare tract for the PV side and an intraparenchymal tract covered with an ePTFE film lining the interior of the stent lumen (Figure 2d). In contrast, the Fluency^®^ is fully covered and does not include an uncovered portion for the PV side (Figure 2c).

In 2017, a new dedicated stent graft known as the VIATORR^®^ TIPS Endoprosthesis with Controlled Expansion (VCX) (W.L. Gore & Associates, Phoenix, AZ, USA) was introduced [23]. The VCX permits adjustment of the diameters of ePTFE-SGs from 8 to 10 mm without any dependence on possible passive dilation (Figure 2e), thus enabling accurate calibration of the PSPG during the TIPS procedure [47]. Collectively, these advances in stent technology have notably enhanced the efficacy of TIPS procedures in managing PH-related complications.

## 3. Bare vs. Covered Stents: Comparative Analysis of Outcomes

To date, four randomized controlled trials (RCTs) [31,32,48,49] have been conducted, including patients who underwent TIPS between 2000 and 2010 (Table 1). Additionally, four systematic reviews [50,51,52,53] have examined the technical and clinical outcomes of ePTFE-SGs versus BMSs. Yang et al. [51] noted that ePTFE-SGs had a superior primary patency rate compared to BMSs in their meta-analysis. Triantafyllou et al. [52] observed not only increased primary shunt patency with ePTFE-SGs but also an improved survival rate and a reduced rebleeding rate, with no significant difference in the incidence of new-onset post-TIPS HE compared to BMS. It is important to acknowledge that both meta-analyses included predominantly non-randomized and retrospective studies, which influenced their methodological uniformity.

Two systematic reviews [51,53] focused exclusively on the four available RCTs. Among these, the recently published Cochrane review by Zhu et al. [53] conducted a thorough analysis and synthesis of the available evidence, with particular emphasis on the impact of dilation diameter as a variable associated with the occurrence of post-TIPS complications. This review evaluated not only shunt patency but also survival rates, post-TIPS HE occurrence, and recurrence rates of variceal bleeding and ascites.

The global certainty of the aforementioned evidence is regarded as low or very low due to several factors, such as a limited number of trials, inadequate sample size, scarcity of events, and an increased risk of bias. 

When examining data from the only RCT [49] that compared 8 mm stents dilated to their nominal diameter, ePTFE-SGs exhibited several advantages over BMSs: a lower mortality rate (RR 0.63, [0.43, 0.92]), reduced incidence of upper gastrointestinal rebleeding (RR 0.54, [0.35, 0.84]), lower recurrence of ascites (RR 0.42, [0.20, 0.87]), and reduced shunt dysfunction (RR 0.42, [0.28, 0.61]). In contrast, there were no significant differences in the incidence of post-TIPS HE (RR 1.10, [0.76, 1.61]). Comparing ePTFE-SGs to BMSs, both with nominal diameters of 10–12 mm [32], 8 mm ePTFE-SGs to 10 mm nominal diameter BMSs [48], and 10 mm nominal diameter ePTFE-SGs to BMSs, both dilated from 8 to 10 mm [31], the ePTFE-SG group exhibited a lower rate of shunt dysfunction (RR 0.50, [0.28, 0.92]). However, the evidence concerning clinical outcomes became more uncertain: the recurrence of ascites remained lower (RR 0.30, [0.11, 0.85]) in the ePTFE-SG group, while no significant differences were detected in terms of mortality rate (RR 0.75, [0.48, 1.16]), upper gastrointestinal bleeding (RR 0.46, [0.15, 1.38]), or post-TIPS HE (RR 0.93, [0.66, 1.30]). 

Qi et al. [51] previously reported comparable outcomes in terms of mortality and shunt dysfunction but did not investigate the impact of different TIPS diameters on the comparison between BMSs and ePTFE-SGs.

Zhu and colleagues [53] concluded by expressing their anticipation of new, larger, and long-term RCTs comparing the use of covered versus bare stents with the same diameter. We acknowledge the methodological limitations of the published studies as highlighted by the Cochrane review. However, the widespread adoption of ePTFE-SGs in studies published in the last two decades coupled with improved placement techniques [24,54,55], enhanced patient selection [13,14,21,22,26], and tailored post-TIPS follow-up management [56], have all provided evidence that supports the therapeutic impact of covered stents for TIPS in patients with PH-related complications. In light of these considerations and based on the current recommendations of the main international guidelines and consensus conferences [16,25,57,58], encouraging the development of trials comparing BMSs to ePTFE-SGs for PH-related complications finds no justification.

## 4. Covered Stents: Technical Tips

TIPS is a complex procedure in the field of interventional radiology. Robust evidence has emerged underscoring the favorable correlation between a higher yearly procedural volume and enhanced patency rates, particularly for ePTFE-SGs [59,60]. Notably, an epidemiological analysis conducted by Sarwar et al. [61] highlighted that patient survival rates improve when performing ≥20 TIPS procedures annually. Subsequently, Buechter et al., in their historical cohort, observed significantly higher rates of TIPS dysfunction when the annual procedure volume fell below this threshold [60]. This highlights the importance of centralizing patients in referral centers with technical expertise, experience in patient selection, and dedicated post-TIPS management [62]. 

As already stated, ePTFE-SGs significantly reduce the rate of TIPS dysfunction. In the Bureau et al. RCT [32], no early thrombosis was observed in the ePTFE-SG group compared to approximately 10% in the BMS group. Another RCT showed that early thrombosis was less frequent in patients receiving ePTFE-SG TIPS than in those receiving BMS (3/63 vs. 10/67) [31]. 

Although the occlusion rate of ePTFE-SG is negligible and anticoagulation is not recommended, a recent survey of 43 German centers showed high heterogeneity, with approximately 50% of centers prescribing anticoagulation after TIPS, regardless of stent type [63]. Prolonged anticoagulation is only recommended for patients with underlying major prothrombotic factors (e.g., FV Leiden, hyperhomocysteinemia, antiphospholipid syndrome, or JAK2 mutation), Budd-Chiari syndrome, or chronic PV thrombosis [64]. 

Outside of these conditions, cases of ePTFE-SG thrombosis in patients with cirrhosis should raise the suspicion of inappropriate TIPS deployment. As reported in an RCT [65] and a subsequent observational study [66], only the subset of patients with concomitant superior mesenteric vein thrombosis and inadequate stent flow due to improper TIPS deployment may benefit from anticoagulation therapy [67]. Indeed, experts have identified two main conditions closely associated with ePTFE-SG dysfunction, both related to technical factors and operator practical knowledge [68].

**Figure 3:**
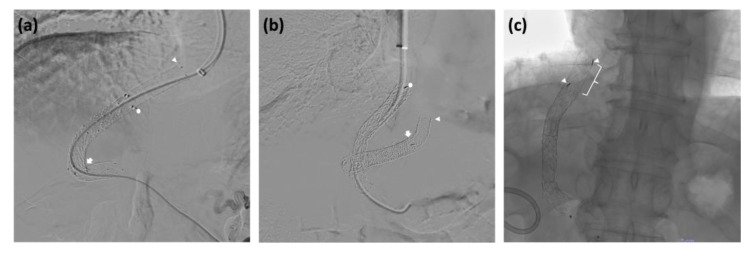
Example of mispositioned TIPS. (**a**) C-shaped TIPS (white arrow), results of coaxial positioning of a second stent graft in order to adequately cover the intraparenchymal and hepatic vein tracts (white dot and arrowhead). (**b**) Placement of a new straight TIPS covering the junction of the HV with the IVC (white dot); the previous short and curved TIPS (white arrow) developed a complete thrombosis despite the deployment of a coaxial longer stent graft (white arrowhead). (**c**) To cover the entire length of the HV beyond the junction with the IVC, a new coaxial stent graft was deployed inside the original TIPS. The white bracket indicates the distance between the original and the coaxial stent graft distal markers (white arrowheads). HV, hepatic vein; IVC, inferior vena cava, TIPS, transjugular intrahepatic portosystemic shunt.

Performing peripheral PV puncture can lead to an excessively curved stent trajectory in the coronal plane, along with a tortuous shift in the sagittal plane. This occurs as the PV and HV branches diverge toward the periphery of the liver parenchyma. This peripheral “C-shaped” TIPS configuration, as depicted in Figure 3, results in an elliptical cross-sectional area that provides significant resistance to blood flow (according to Poiseuille’s law) and increases the risk of thrombosis in the stent. To achieve an optimal configuration, it is essential to position the ePTFE-SG centrally, linking the right branch of the PV and the main right HV. This determines a “straight” TIPS configuration characterized by a circular cross-sectional area, more in line with the diameter of the dilation balloon [69]. 

Well-established since the early 1990s [70], an ultrasound-guided puncture of the PV ensures a fast and dynamic assessment of the vascular anatomy, leading to better technical results.

In cases of ePTFE-SG dysfunction resulting from a “C-shaped” TIPS, percutaneous transluminal angioplasty may prove ineffective in rectifying the shunt. In such circumstances, it may be necessary to place a new ePTFE-SG through a new straight intraparenchymal channel. With these considerations in mind, combined percutaneous trans-hepatic or trans-splenic portal access may be considered when anatomic changes, such as cavernomatous PV transformation, result in a limited PV landing zone for the transjugular approach [71]. These technical solutions typically allow for more precise PV puncture in complex scenarios, facilitating the creation of a straight connection with the HV.

Additional factors contributing to ePTFE-SG dysfunction are linked to mispositioning and/or improper deployment of the endoprosthesis. It is critical to ensure that the length of the ePTFE-SG is sufficient to cover the entire distance from the PV wall to the HV (the covered section), up to its convergence with the IVC (Figure 3). Failure to provide such coverage leads to the development of endothelial hyperplasia due to shear stress within the non-stented segment of the HV [72,73]. This results in stenosis, increased PSPG, and, ultimately, TIPS dysfunction. 

In fact, a deep learning model has identified that a distance greater than or equal to 6 mm from the distal end of the ePTFE-SG to the hepatocaval junction is a strong predictor of the recurrence of post-TIPS PH-related complications [74]. Likewise, the gold marker band in the VTS and VCX (Figure 2) should be positioned to align with the PV wall. Excessive retraction of dedicated ePTFE-SGs may cause a portion of the bare section to drag within the intraparenchymal tract, increasing the risk of intimal hyperplasia and subsequent shunt dysfunction. Conversely, deploying these ePTFE-SGs without retracting the covered section to the point of passage through the PV wall may hamper venous blood flow to or from the PV branches. 

It remains imperative to ensure that the TIPS procedure does not impair a potential future liver transplantation. Therefore, the ePTFE-SG should not extend toward the convergence of the splenic and superior mesenteric veins, as this could disrupt the prime location for surgical PV anastomosis [73]. Similarly, placement of the ePTFE-SG within the right atrium may complicate surgical anastomosis of the IVC [75].

It is noteworthy that stent migration is more prevalent with ePTFE-SGs than with BMSs, although it remains a relatively infrequent complication [76]. The primary causes of stent migration are associated with procedural errors, particularly the premature retraction of the dilatation balloon before its complete deflation. In light of the increasing adoption of under-dilation techniques, it is necessary to emphasize that the diameter of the parenchymal channel must not exceed the final dilation caliber of the endoprosthesis. Indeed, this will prevent self-expansion of the device.

The superior primary patency rate shown by ePTFE-SGs determines their cost-effectiveness for TIPS placement despite their higher cost compared to BMSs [31]. This is mainly due to their efficacy in reducing clinical relapses of PH-related complications and in preventing further decompensation [26,77,78]. These effects significantly curtail the risk of hospitalization, thus mitigating healthcare burdens and reducing costs. Nevertheless, the use of BMSs may emerge as a cost-effective alternative in liver transplant candidates with an estimated waiting time of less than 3–4 months. Within this narrow timeframe, the primary patency rate may be expected to exceed 85% [31,32].

## 5. The Technical Evolution of TIPS Does Not Stand Alone in Improving Outcomes

**Figure 4:**
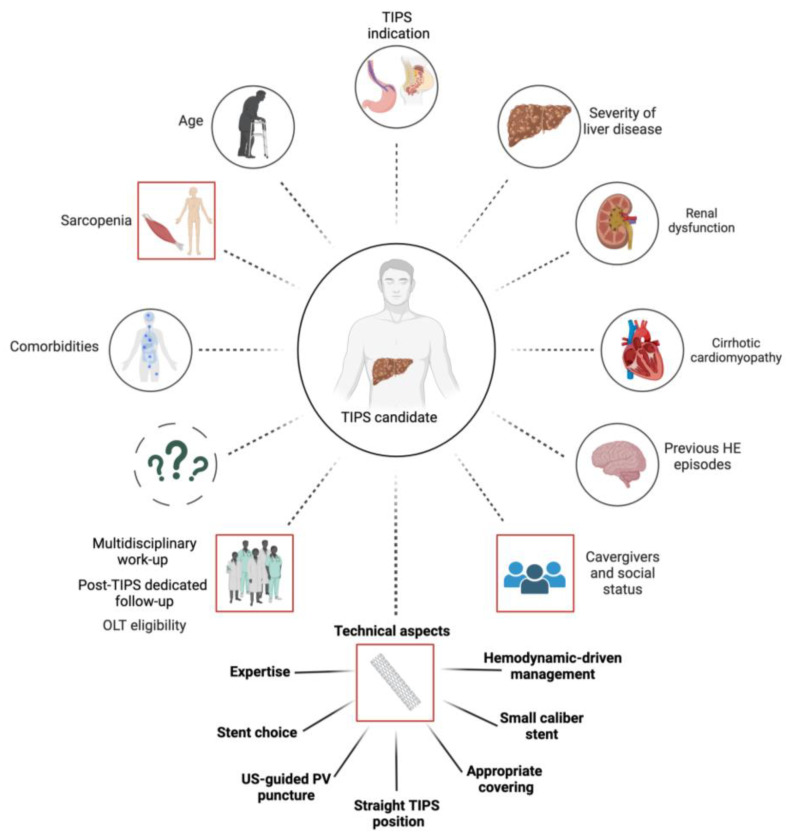
Determinants of TIPS outcomes. Closed circle = intrinsic factors; dashed circle = unknown factors; red squared = extrinsic factors. Abbreviations: HE, hepatic encephalopathy; OLT, orthotopic liver transplantation; PV, portal vein; TIPS, transjugular intrahepatic portosystemic shunt; US, ultrasound. (Created with BioRender.com).

Figure 4 depicts the intricate network of interactions and the subsequent complexity involved in tailoring the management of candidates for TIPS placement. Currently, the personalized application of TIPS remains challenging due to numerous factors that can influence the outcome. Some of them are partially understood, others are still unknown [79,80,81,82]. 

Although the issue of shunt patency has been resolved with the introduction of ePTFE-SGs, the primary barriers to expanding the use of TIPS are the insufficient understanding of both its potential to reverse the physiopathology of portal hypertension and the mechanisms responsible for shunt-related complications, including post-TIPS HE, liver failure, and heart failure [83]. Patients with decompensated cirrhosis present with a multifaceted clinical scenario, marked by alterations in both splanchnic and systemic hemodynamics, which are associated with increased systemic inflammation and multiorgan dysfunction [83,84]. Understanding the complex mechanisms leading to decompensated cirrhosis is inherently challenging. 

Further complexity is added by factors such as aging demographics [82], increased comorbidities (particularly cardiovascular) [80], and the growing prevalence of non-alcoholic steatohepatitis [85]. Consequently, deepening our comprehension of heart and other organs damage and the systemic and splanchnic hemodynamics before and after TIPS placement assumes paramount importance [86]. Such knowledge will facilitate the identification of predictive markers of outcomes, enable more effective management of shunt-related complications, and enhance the integration of synergistic drugs as adjunctive treatments [68,87]. Overcoming these limitations represents the next frontier in advancing the field of TIPS.

### 5.1. Hemodynamic Targets

Updated and methodologically rigorous research is needed to establish post-TIPS PSPG targets in light of contemporary epidemiological realities, which result from etiological shifts and demographic changes. Trials conducted over two decades ago provide a significant portion of the evidence. However, it is challenging to apply their findings to the present scenario.

These studies predominantly employed BMSs directly dilated to a nominal diameter of 10 to 12 mm. Initial propositions, such as Casado et al.’s recommendation of a post-TIPS PSPG <12 mmHg as the hemodynamic target for TIPS, regardless of the indication, still lack firm validation [88]. In particular, attempts to correlate post-TIPS PSPG with ascites control have yielded inconsistent outcomes and no definitive threshold [9,89,90,91]. Therefore, the debate on the ideal PSPG reduction for the treatment of ascites is still ongoing. 

Recent studies have shown that TIPS created with ePTFE-SGs can achieve favorable clinical outcomes in both ascites control [24] and variceal bleeding prevention [24,54,92] without requiring a reduction in PSPG below conventional thresholds of <12 mmHg. Moreover, the Baveno VII consensus [25] suggests that immediate post-TIPS PSPG measurements may not accurately reflect the true value due to factors such as anesthesia, vasoactive medications, pain, and latency in splanchnic hemodynamic adaptations [93]. Experts advocate for repeated post-TIPS PSPG assessments under hemodynamically stable, non-sedated conditions to enhance accuracy. 

New RCTs that use the latest generation of dedicated ePTFE-SGs and incorporate gold standard and serial hemodynamic evaluations after TIPS [25] are necessary to validate the <12 mmHg threshold for bleeding indications and to investigate the significance and effectiveness of a PSPG-based approach in the ascites setting.

### 5.2. Shunt-Related Complications

The objective of improving TIPS focuses on identifying the least amount of shunting intervention required to manage PH-related complications while minimizing the risk of shunt-related adverse events. Excessive shunting of portal blood flow constitutes the primary driver of post-TIPS complications. When portal blood shunting reaches approximately 70% or more, an expansion of the hepatic artery bed will compensate for the decreased perfusion of the liver [94,95]. This renders liver perfusion reliant on cardiac performance, and an insufficient inotropic response may result in hepatic ischemia and subsequent liver failure [96,97]. 

Similarly, a sudden rise in cardiac preload due to diastolic dysfunction will lead to heart failure and hepatic vein congestion. Recent guidelines advocate for a thorough evaluation of cardiac function in TIPS candidates to detect underlying cardiomyopathies, although the most suitable diagnostic method and marker remain to be defined [80]. Strategies involving baseline and post-TIPS right heart catheterization, especially in high-risk patients, along with the use of smaller caliber ePTFE-SGs, may represent an effective strategy to prevent cardiac and liver complications [69].

The most common complication following TIPS is HE, occurring in approximately 35–50% of cases [10,26,98]. While the majority of cases are episodic and can be triggered by various factors such as dehydration, infection, or constipation, with rapid symptom resolution upon addressing the precipitant, 5–10% of instances manifest as recurrent or persistent and do not respond adequately to therapy [99,100]. Previous episodes of HE [58], severe liver dysfunction [6,10,22], aging [82], sarcopenia [101,102], elevated creatinine levels, and hyponatremia [82,103] stand out as major patient predisposing factors for post-TIPS HE. Awareness of these correlations enables better patient selection, the application of preventive interventions, and closer monitoring of high-risk individuals [104]. 

In addition to these patient-related factors, stent diameter is directly linked to the risk of post-TIPS HE: larger shunts are associated with a higher risk of this event occurring [24,54].

### 5.3. Choice of Stent Nominal Diameter

Several research studies have examined the postoperative clinical efficacy, hemodynamic effects, and occurrence of HE with various nominal diameters, resulting in divergent outcomes. Two studies, an early interrupted RCT by Riggio et al. [105], which enrolled 45 patients with either prevention of variceal rebleeding or refractory ascites as indications, and the retrospective study by Miraglia et al. [106], which included 171 patients with refractory ascites, compared patients who received ePTFE-SG with nominal diameters of 10 mm or 8 mm. Patients who received the 8 mm ePTFE-SG showed comparable rates of HE. However, their clinical efficacy was lower owing to the recurrence of ascites. Notably, it is important to mention that the 10 mm and 8 mm groups in both studies presented an immediate mean post-TIPS PSPG below 10 mmHg, which makes it difficult to reconcile their results of clinical efficacy. Additionally, in the study by Miraglia et al., it was observed that 50% of patients requiring TIPS revision during follow-up for ascites recurrence had a PSPG below the level of 12 mmHg [106]. In contrast, a more recent RCT involving 127 patients reported that 8 mm ePTFE-SG is as effective as the 10 mm version in preventing variceal rebleeding. Moreover, 8 mm ePTFE-SG reduced the incidence of post-TIPS HE, regardless of the post-TIPS PSPG [54]. Luo et al. [107] further corroborated these results in a retrospective, propensity score matched cohort of 62 patients who underwent TIPS for variceal bleeding, exhibiting a lower risk of HE in the 8 mm group.

While none of the reported studies indicated differences in overall survival, Trebicka et al. [108] found that 41 patients who received 8 mm ePTFE-SG had enhanced transplant-free survival in comparison to the 41 that received a 10 mm ePTFE-SG after being matched for age, MELD score, and serum bilirubin. Nonetheless, the 8 mm group still had a significantly lower Child-Pugh class and a higher frequency of indications for variceal bleeding compared to the matched groups.

A meta-analysis examining all of the above mentioned studies concluded that the use of ePTFE-SG with an 8 mm diameter results in a lower risk of HE, a similar risk of variceal rebleeding, a higher rate of stent dysfunction, and an improvement in overall survival after 1 and 3 years [109]. The results of this meta-analysis are affected by population heterogeneity, small sample sizes, and the inclusion of retrospective cohort studies.

### 5.4. The Underdilation Strategy

Positioning small-caliber ePTFE-SGs has the potential to expand the use of TIPS by improving its safety profile [68,110]. However, limited data exist on the impact of utilizing ePTFE-SGs underdilated to diameters smaller than the nominal one. Table 2 summarizes previous studies examining the behavior of the 10 mm VTS underdilated to 8 mm, suggesting that it would spontaneously dilate over time [54,58,93,111].

In contrast, the sole study [24] that assessed the behavior of underdilated VTS at diameters inferior to 8 mm revealed that no ePTFE-SGs reached the nominal diameter, and only a small percentage spontaneously dilated by 1 mm over time. It is worth noting that, unlike previous studies that relied on less precise techniques like ultrasound [114] or digital angiography [113], or focused solely on evaluating stent diameter/area within the intraparenchymal tract [75,112], Schepis et al. [24] employed computed tomography (CT) to measure the average maximum inner diameter of the ePTFE-SGs at several predetermined sites. They found no significant self-expansion of the ePTFE-SGs at the passages through the PV and HV walls. Moreover, the aforementioned study and a later Chinese case–control study [115] provided evidence that underdilation to 6 mm may decrease the occurrence of post-TIPS HE without any differences in the risk of recurrent bleeding, ascites, or ePTFE-SG thrombosis.

The introduction of VCX, which does not spontaneously expand beyond 8 mm [23,47], presents potential advantages for the stability of the underdilated inner diameter [55]. In a preliminary feasibility analysis conducted by our research group [116], we compared the behavior of 60 TIPS that were underdilated to a diameter of 6 mm (20 VCX, 20 VTS 8 mm nominal diameter, and 20 VTS 10 mm nominal diameter) using CT scans performed more than 1 month after TIPS placement. We evaluated the average maximal inner diameters at the PV and HV walls, as previously reported [24]. Our findings indicated that VCX maintained a dilatation diameter similar to VTS 8 mm at both the PV wall (6.2 mm vs. 6.1 mm, *p*-value 0.471) and HV wall (6.0 mm vs. 6.3 mm, *p*-value 0.196) but significantly better than VTS 10 mm (PV wall: 6.2 mm vs. 6.8 mm, *p*-value 0.044; HV wall: 6.0 mm vs. 6.9 mm, *p*-value 0.004). The development of ePTFE-SGs with CX technology spanning a wider range of diameters, such as 6–10 mm, may allow more precise modulation of the PSPG reduction according to each patient’s clinical response.

## 6. Conclusions

In summary, the TIPS procedure has evolved significantly over the years. Technical advances in stent technology have been instrumental in establishing TIPS as a central therapy for the treatment of PH-related complications. The shift from BMSs to dedicated ePTFE-SGs was a pivotal step, leading to significant improvements in patency rates. The use of small-caliber TIPS has the potential to improve procedural control and significantly reduce shunt-related complications. However, a personalized approach is required to position TIPS at the forefront of the modern treatment of PH.

## Figures and Tables

**Table 1:** Characteristics of RCTs comparing BMSs to ePTFE-SGs.

Study	Design	Groups/Pts	Stents/Nominal Diameter (mm)	Indication/*n*	Etiology of Cirrhosis/*n*	Follow-Up (Days)	Study End-Points	Results
Bureau et al., 2007 [32]	Multicente, unblinded	BMS 41	Memotherm Flexx^®^ (BARD), Wallstent^®^ (Boston), Luminex^®^ (BARD), Sinus Stent^®^ (MEDCARE)/NA ^#^	RA, 12; AVB, 14; Prevention of rebleeding, 15.	Alcohol, 22.	430 ± 322	1st—shunt dysfunction rate. 2nd—relapse of ascites (need for LVP) or gastrointestinal bleeding; number of revisions for shunt patency; rates of complication, HE and survival.	ePTFE-SG improved primary patency, reduced clinical relapses and post-TIPS HE rate. No significant difference in survival rate.
		ePTFE-SG 39	Viatorr^®^ (GORE)/NA ^#^	RA, 20; AVB, 9; Prevention of rebleeding, 10.	Alcohol, 22.	585 ± 438		
Huang et al., 2010 [48]	Single-center, unblinded	BMS 30	Wallstents^®^ (Boston)/10	PH-related bleeding, 26; RA or hydrothorax, 4.	Viral, 29; Alcohol, 1.	249 ± 132	The role of Doppler US in quantitative assessment of shunt function and the usefulness of routine US follow-up of ePTFE-SGs.	Routine US surveillance may not be necessary for ePTFE-SG. ePTFE-SG improved primary patency rate.
		ePTFE-SG 30	Fluency^®^ (BARD)/8	PH-related bleeding, 25; RA or hydrothorax, 5.	Viral, 28; Alcohol, 2.	186 ± 117		
Perarnau et al., 2014 [31]	Multicenter, single-blind	BMS 66	Luminexx^®^ (BARD), Palmaz Genesis^®^ (Cordis), Smart^®^ (Cordis), Wallstent^®^ (Boston), Zilver^®^ (Cook)/NA ^#^	Prevention of rebleeding, 22; RA, 46; Hydrothorax, 6 ^†^.	Alcohol, 61; Viral, 8; NASH, 1; Others, 2.	654 ^§^ (IQR 171–723)	1st—shunt dysfunction rate. 2nd—early complications (≤1 month); symptoms recurrence; rate of HE/ disabling chronic; quality of life; early (≤1 month) and late mortality.	ePTFE-SG improved primary patency and reduced clinical relapses rate. No significant difference in post-TIPS HE and survival rate.
		ePTFE-SG 71	Fluency^®^ (BARD), Fluency^®^ + BMs, Viatorr^®^ (GORE)/10	Prevention of rebleeding, 20; RA, 54; Hydrothorax, 3 ^†^.	Alcohol, 52; Viral, 10; NASH, 6; Others, 1.	708 ^§^ (IQR 420–723)		
Wang et al., 2016 [49]	Single-center, double-blind	BMS 131	Smart^®^ (Cordis)/8	PH-related bleeding, 122; RA, 22 ^†^.	Viral, 102; Others, 25.	NA (5 year)	Restenosis/occlusion rate; recurrence of gastrointestinal bleeding, ascites/ hydrothorax; rate of secondary interventional therapy, HE and survival.	ePTFE-SGs improved both primary and secondary patency rate, and reduced clinical relapses rate.ePTFE-SG significantly improved long-term survival. No significant difference in post-TIPS HE rate.
		ePTFE-SG 127	Fluency^®^ (BARD)/8	PH-related bleeding, 123; RA, 20.	Viral, 104; Alcohol, 27.			

AVB, acute variceal bleeding; BMSs, bare metal stents; ePTFE-SGs, expanded polytetrafluoroethylene-stent grafts; HE, hepatic encephalopathy; LVP, large volume paracentesis; NASH, non-alcoholic steatohepatitis; PH, portal hypertension; RA, refractory ascites; TIPS, transjugular intrahepatic portosystemic shunt; US, ultrasound. ^#^ The nominal diameter of stents is not specified, and shunt diameter is only expressed as mean ± SD for each group. ^†^ TIPS indications may be more than one. ^§^ Median follow-up.

**Table 2:** Studies investigating the performance of VTS ePTFE-SGs when underdilated to a caliber smaller than their nominal diameters.

Study	Design/Pts	Nominal Ø (mm)	Under-Dilation (mm)	Imaging	Sites of Measurement	Type of Measurement	Assessment of Clinical Outcomes
Gaba et al., 2015 [75]	Retro; 61	10	8	CT	IP tract	Cross-sectional Ø from the midportion of the metal wall	No
Pieper et al., 2015 [112]	Retro; 29	8 (*n* = 1) 10 (*n* = 28)	7 (*n* = 1) 8 (*n* = 28)	CT	PVW, IP, HVW	Cross-sectional area at each site	No
Borghol et al., 2016 [113]	Retro; 16	10	8	Digital angiography	Gold ring marker-IP-HVW	Mean internal stent Ø	No
Pieper et al., 2017 [114]	Prosp; 20	10	8	US	IP tract excluding PVW and HVW	Mean of 10 Ø measurements from the midportion of the wall reflex	No
Schepis et al., 2018 [24]	Retro; 226 Prosp: 142	8–10	6	CT	PVW and HVW	Mean of the largest cross-sectional inner Ø	Yes

Ø = diameter; Abbreviations: CT, computed tomography; HVW, hepatic vein wall; IP, intraparenchymal; Pts, patients; Prosp, prospective; PVW, portal vein wall; Retro, retrospective; US, ultrasound.

## Data Availability

Data sharing not applicable.
